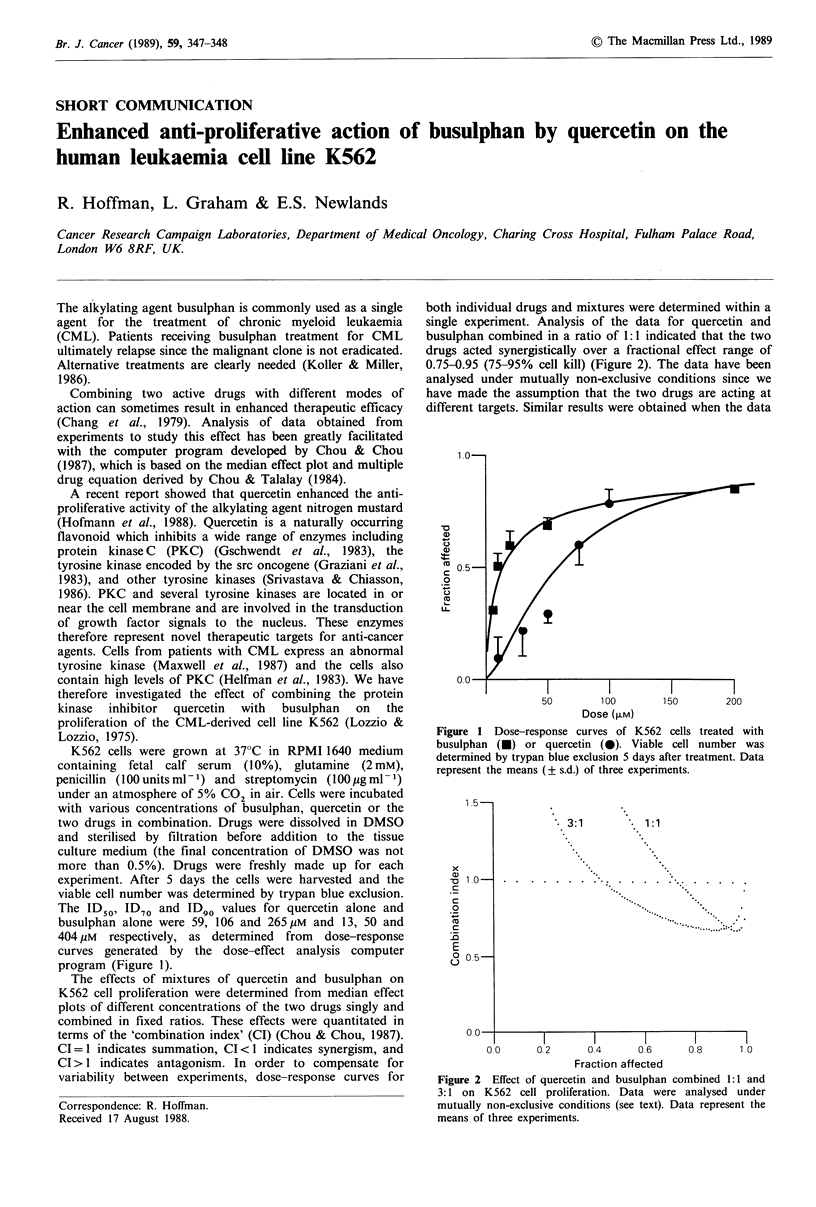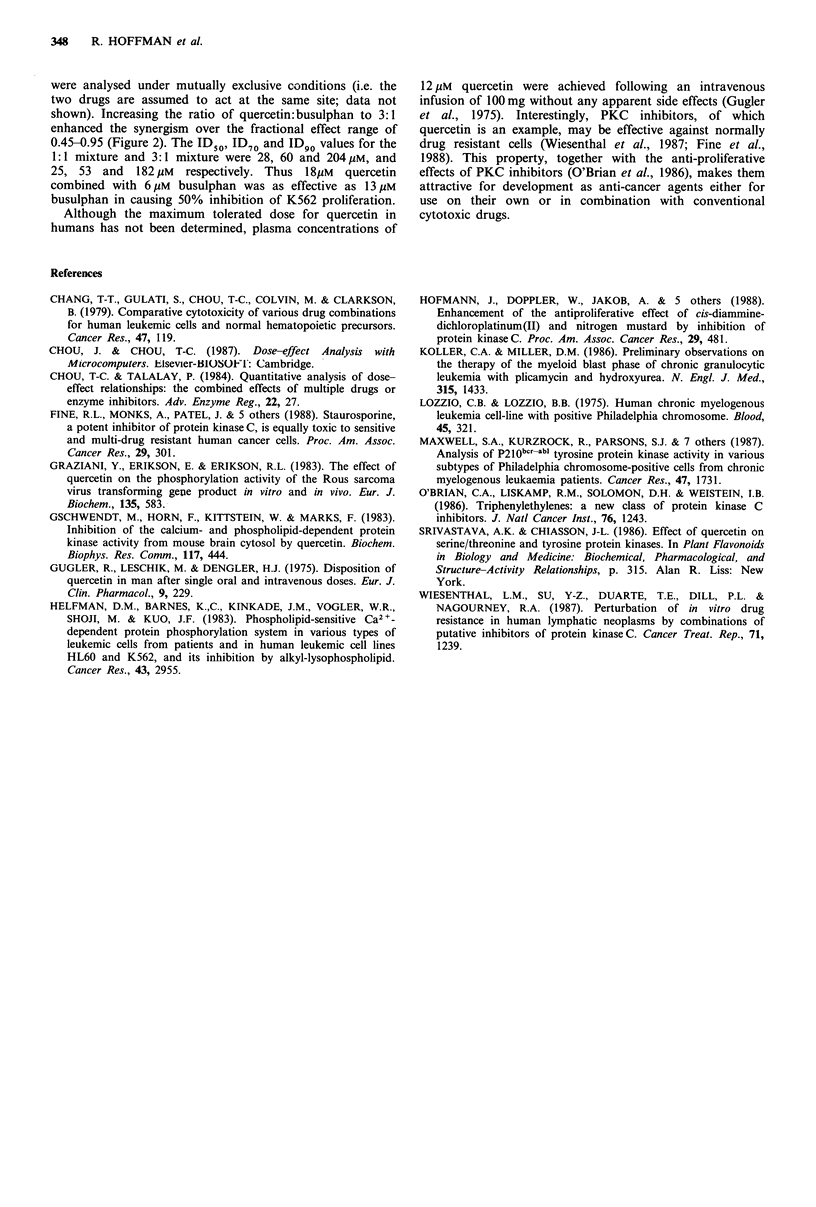# Enhanced anti-proliferative action of busulphan by quercetin on the human leukaemia cell line K562.

**DOI:** 10.1038/bjc.1989.68

**Published:** 1989-03

**Authors:** R. Hoffman, L. Graham, E. S. Newlands

**Affiliations:** Cancer Research Campaign Laboratories, Department of Medical Oncology, Charing Cross Hospital, London, UK.


*Br. J. Cancer* (1989), **59**, 347–348 © The Macmillan Press Ltd., 1989

SHORT COMMUNICATION

### Enhanced anti-proliferative action of busulphan by quercetin on the human leukaemia cell line K562

R. Hoffman, L. Graham & E.S. Newlands

*Cancer Research Campaign Laboratories, Department of Medical Oncology, Charing Cross Hospital, Fulham Palace Road, London W6 8RF, UK.*

The alkylating agent busulphan is commonly used as a single agent for the treatment of chronic myeloid leukaemia (CML). Patients receiving busulphan treatment for CML ultimately relapse since the malignant clone is not eradicated. Alternative treatments are clearly needed (Koller & Miller, 1986).

Combining two active drugs with different modes of action can sometimes result in enhanced therapeutic efficacy (Chang *et al.*, 1979). Analysis of data obtained from experiments to study this effect has been greatly facilitated with the computer program developed by Chou & Chou (1987), which is based on the median effect plot and multiple drug equation derived by Chou & Talalay (1984).

A recent report showed that quercetin enhanced the anti-proliferative activity of the alkylating agent nitrogen mustard (Hofmann *et al.*, 1988). Quercetin is a naturally occurring flavonoid which inhibits a wide range of enzymes including protein kinase C (PKC) (Gschwendt *et al.*, 1983), the tyrosine kinase encoded by the src oncogene (Graziani *et al.*, 1983), and other tyrosine kinases (Srivastava & Chiasson, 1986). PKC and several tyrosine kinases are located in or near the cell membrane and are involved in the transduction of growth factor signals to the nucleus. These enzymes therefore represent novel therapeutic targets for anti-cancer agents. Cells from patients with CML express an abnormal tyrosine kinase (Maxwell *et al.*, 1987) and the cells also contain high levels of PKC (Helfman *et al.*, 1983). We have therefore investigated the effect of combining the protein kinase inhibitor quercetin with busulphan on the proliferation of the CML-derived cell line K562 (Lozzio & Lozzio, 1975).

K562 cells were grown at 37°C in RPMI 1640 medium containing fetal calf serum (10%), glutamine (2 mM), penicillin ($100\,units\,ml^{-1}$) and streptomycin ($100\,\mu g\,ml^{-1}$) under an atmosphere of 5% $CO_2$ in air. Cells were incubated with various concentrations of busulphan, quercetin or the two drugs in combination. Drugs were dissolved in DMSO and sterilised by filtration before addition to the tissue culture medium (the final concentration of DMSO was not more than 0.5%). Drugs were freshly made up for each experiment. After 5 days the cells were harvested and the viable cell number was determined by trypan blue exclusion. The $ID_{50}$, $ID_{70}$ and $ID_{90}$ values for quercetin alone and busulphan alone were 59, 106 and 265 $\mu$M and 13, 50 and 404 $\mu$M respectively, as determined from dose–response curves generated by the dose–effect analysis computer program (Figure 1).

The effects of mixtures of quercetin and busulphan on K562 cell proliferation were determined from median effect plots of different concentrations of the two drugs singly and combined in fixed ratios. These effects were quantitated in terms of the 'combination index' (CI) (Chou & Chou, 1987). CI = 1 indicates summation, CI < 1 indicates synergism, and CI > 1 indicates antagonism. In order to compensate for variability between experiments, dose–response curves for both individual drugs and mixtures were determined within a single experiment. Analysis of the data for quercetin and busulphan combined in a ratio of 1:1 indicated that the two drugs acted synergistically over a fractional effect range of 0.75–0.95 (75–95% cell kill) (Figure 2). The data have been analysed under mutually non-exclusive conditions since we have made the assumption that the two drugs are acting at different targets. Similar results were obtained when the data

**Figure 1** Dose–response curves of K562 cells treated with busulphan (■) or quercetin (●). Viable cell number was determined by trypan blue exclusion 5 days after treatment. Data represent the means (± s.d.) of three experiments.

**Figure 2** Effect of quercetin and busulphan combined 1:1 and 3:1 on K562 cell proliferation. Data were analysed under mutually non-exclusive conditions (see text). Data represent the means of three experiments.

Correspondence: R. Hoffman.
Received 17 August 1988.

were analysed under mutually exclusive conditions (i.e. the two drugs are assumed to act at the same site; data not shown). Increasing the ratio of quercetin:busulphan to 3:1 enhanced the synergism over the fractional effect range of 0.45–0.95 (Figure 2). The $ID_{50}$, $ID_{70}$ and $ID_{90}$ values for the 1:1 mixture and 3:1 mixture were 28, 60 and 204 $\mu$M, and 25, 53 and 182 $\mu$M respectively. Thus 18 $\mu$M quercetin combined with 6 $\mu$M busulphan was as effective as 13 $\mu$M busulphan in causing 50% inhibition of K562 proliferation.

Although the maximum tolerated dose for quercetin in humans has not been determined, plasma concentrations of 12 $\mu$M quercetin were achieved following an intravenous infusion of 100 mg without any apparent side effects (Gugler *et al.*, 1975). Interestingly, PKC inhibitors, of which quercetin is an example, may be effective against normally drug resistant cells (Wiesenthal *et al.*, 1987; Fine *et al.*, 1988). This property, together with the anti-proliferative effects of PKC inhibitors (O'Brian *et al.*, 1986), makes them attractive for development as anti-cancer agents either for use on their own or in combination with conventional cytotoxic drugs.

### References

CHANG, T-T., GULATI, S., CHOU, T-C., COLVIN, M. & CLARKSON, B. (1979). Comparative cytotoxicity of various drug combinations for human leukemic cells and normal hematopoietic precursors. *Cancer Res.*, **47,** 119.

CHOU, J. & CHOU, T-C. (1987). *Dose-effect Analysis with Microcomputers.* Elsevier-BIOSOFT: Cambridge.

CHOU, T-C. & TALALAY, P. (1984). Quantitative analysis of dose–effect relationships: the combined effects of multiple drugs or enzyme inhibitors. *Adv. Enzyme Reg.*, **22,** 27.

FINE, R.L., MONKS, A., PATEL, J. & 5 others (1988). Staurosporine, a potent inhibitor of protein kinase C, is equally toxic to sensitive and multi-drug resistant human cancer cells. *Proc. Am. Assoc. Cancer Res.*, **29,** 301.

GRAZIANI, Y., ERIKSON, E. & ERIKSON, R.L. (1983). The effect of quercetin on the phosphorylation activity of the Rous sarcoma virus transforming gene product *in vitro* and *in vivo*. *Eur. J. Biochem.*, **135,** 583.

GSCHWENDT, M., HORN, F., KITTSTEIN, W. & MARKS, F. (1983). Inhibition of the calcium- and phospholipid-dependent protein kinase activity from mouse brain cytosol by quercetin. *Biochem. Biophys. Res. Comm.*, **117,** 444.

GUGLER, R., LESCHIK, M. & DENGLER, H.J. (1975). Disposition of quercetin in man after single oral and intravenous doses. *Eur. J. Clin. Pharmacol.*, **9,** 229.

HELFMAN, D.M., BARNES, K.,C., KINKADE, J.M., VOGLER, W.R., SHOJI, M. & KUO, J.F. (1983). Phospholipid-sensitive $Ca^{2+}$-dependent protein phosphorylation system in various types of leukemic cells from patients and in human leukemic cell lines HL60 and K562, and its inhibition by alkyl-lysophospholipid. *Cancer Res.*, **43,** 2955.

HOFMANN, J., DOPPLER, W., JAKOB, A. & 5 others (1988). Enhancement of the antiproliferative effect of *cis*-diammine-dichloroplatinum(II) and nitrogen mustard by inhibition of protein kinase C. *Proc. Am. Assoc. Cancer Res.*, **29,** 481.

KOLLER, C.A. & MILLER, D.M. (1986). Preliminary observations on the therapy of the myeloid blast phase of chronic granulocytic leukemia with plicamycin and hydroxyurea. *N. Engl. J. Med.*, **315,** 1433.

LOZZIO, C.B. & LOZZIO, B.B. (1975). Human chronic myelogenous leukemia cell-line with positive Philadelphia chromosome. *Blood*, **45,** 321.

MAXWELL, S.A., KURZROCK, R., PARSONS, S.J. & 7 others (1987). Analysis of $P210^{bcr-abl}$ tyrosine protein kinase activity in various subtypes of Philadelphia chromosome-positive cells from chronic myelogenous leukaemia patients. *Cancer Res.*, **47,** 1731.

O'BRIAN, C.A., LISKAMP, R.M., SOLOMON, D.H. & WEISTEIN, I.B. (1986). Triphenylethylenes: a new class of protein kinase C inhibitors. *J. Natl Cancer Inst.*, **76,** 1243.

SRIVASTAVA, A.K. & CHIASSON, J-L. (1986). Effect of quercetin on serine/threonine and tyrosine protein kinases. In *Plant Flavonoids in Biology and Medicine: Biochemical, Pharmacological, and Structure-Activity Relationships*, p. 315. Alan R. Liss: New York.

WIESENTHAL, L.M., SU, Y-Z., DUARTE, T.E., DILL, P.L. & NAGOURNEY, R.A. (1987). Perturbation of *in vitro* drug resistance in human lymphatic neoplasms by combinations of putative inhibitors of protein kinase C. *Cancer Treat. Rep.*, **71,** 1239.